# Oberst's Method of Inducing Local Anæsthesia

**Published:** 1903-09-19

**Authors:** 


### OBERST'S METHOD OF INDUCING LOCAL ANÆSTHESIA.

There are times when the knowledge of how to produce local anæsthesia is of the greatest service, nor is it necessary to give illustrations on this point. But most of the ordinary methods of rendering a small portion of the body anæsthetic are liable to fail when one comes to deal with tissues into which there is much inflammatory exudation or where dense structures such as bones and ligaments are to be interfered with. It is in just these cases that Oberst's method may be of value. Oberst found that a finger could be made quite insensitive if a tourinquet were applied to its base, and a solution of cocaine injected round the digital nerves. His observations were published in 1890, and Struthers[1] states that since then the method has come into general use on the Continent for amputating fingers, removing nails, opening whitlows, and for all operations on the fingers and toes in which anæsthesia of the part is necessary. To anæsthetise a finger a rubber tourniquet is applied at its base—not too forcibly, for the circulation in a finger is easily arrested, and if the tourniquet be too tight the pressure will cause discomfort and may damage the nerves—and from 5 to 10 minims of a 1 per cent. solution of cocaine are injected directly into the subcutaneous tissue of the finger round each digital nerve, distal to the tourniquet, four punctures being thus required. In 10 minutes the finger will be completely anæsthetic, and even amputation may be painlessly performed. Anæsthesia lasts as long as the tourniquet is left on and for some minutes after it has been removed. The pain caused by the tourniquet, and the difficulty of cocainising large nerves, limits the application of the method, for the most part, to operations upon the fingers and toes.

[1] Edinburgh Medical Journal, Aug. 1903.

